# Mullerian anomalies: revisiting imaging and classification

**DOI:** 10.1186/s13244-024-01879-2

**Published:** 2025-02-17

**Authors:** Rashmi Dixit, Chitty Suvarna Duggireddy, Gaurav Shanker Pradhan

**Affiliations:** Department of Radiodiagnosis, Maulana Azad Medical College and associated Lok Nayak Hospital, New Delhi, India

**Keywords:** Mullerian duct anomalies, Magnetic resonance imaging, Infertility, Reproductive medicine, Embryology

## Abstract

**Abstract:**

Mullerian duct anomalies (MDA) are a group of uncommon but treatable causes of infertility and pregnancy complications. This review describes the embryology, American Society of Reproductive Medicine (ASRM) classification 2021, and corresponding imaging features of MDA. The three phases of embryological development of Mullerian duct structures are described. The main emphasis is on the ASRM 2021 classification of MDA into nine descriptive categories, while the European Society of Human Reproduction and Embryology and the European Society for Gynecologic Endoscopy (ESHRE/ESGE) classification is also briefly described where necessary. MRI imaging features of MDA along with the acquisition techniques are discussed in detail, as MRI is the ideal imaging modality for MDA diagnosis. In addition, the current role of imaging modalities such as hysterosalpingography and ultrasound including 3D transvaginal ultrasound is also elucidated. The review aims to revisit the MRI imaging features of Mullerian anomalies and reiterates that an accurate description of each anomaly and precise communication with clinicians is the priority rather than rigidly fitting the anomaly into one particular category.

**Critical relevance statement:**

The ASRM 2021 classification of Mullerian anomalies has re-defined the criteria for an arcuate uterus. Radiologists must know of the new classification and imaging features and try to describe each anomaly accurately rather than forcefully fitting an anomaly into a definite category.

**Key Points:**

- MDA has an important role in infertility and pregnancy complications.
- Knowledge of imaging features helps radiologists aid patient management; MRI is the preferred imaging modality for MDAs.
- An accurate MRI-based description of Mullerian anomalies is crucial, avoiding the pitfalls of rigid categorization.

**Graphical Abstract:**

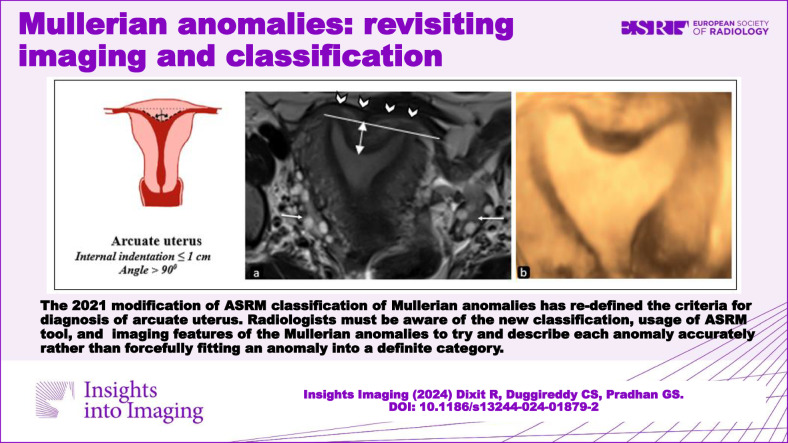

## Introduction

A disturbance in the development of the paired Mullerian (paramesonephric) ducts, which normally give rise to the uterus, fallopian tubes, cervix, and upper vagina, results in Mullerian duct anomalies (MDAs) [1]. MDAs are common, with a prevalence of 5.5% in the general population [1]. Higher prevalences of ~8% and ~13.3–24.5% are reported among women with infertility and miscarriages, respectively [2]. These anomalies are associated with reproductive problems such as primary amenorrhea, endometriosis, infertility, and obstetric complications such as ectopic pregnancy, miscarriage, preterm labor, malposition, and postpartum hemorrhage, causing great anxiety to patients. It is important that these be identified and appropriately classified for the best clinical outcome. This review will mainly focus on the MR imaging appearances of the common and some unclassified MDAs.

Mullerian anomalies were initially classified into seven classes by the American Fertility Society (AFS) in 1988. The latest modification of Mullerian anomalies by the American Society of Reproductive Medicine (ASRM) in 2021 is the most widely used clinically [3]. Another classification system, given by the European Society of Human Reproduction and Embryology and the European Society for Gynecologic Endoscopy (ESHRE/ESGE) in 2013, is also commonly used. This review aims to compare the ASRM 2021 and ESHRE/ESGE 2013 classification of MDAs especially regarding the septate uterus, which is often overdiagnosed by the latter classification system, as concluded by several authors [4]. In addition, the fact that the clinical implication of this diagnosis in patients’ reproductive outcomes has not been fully evaluated under the ESHRE/ESGE classification has also been highlighted in several reports [4]. Radiologists and gynecologic colleagues must be aware of the important entities in these two classification systems, the differences between the two, and their implications in management. This review will focus on these important aspects.

## Embryology

**Fig. 1:**
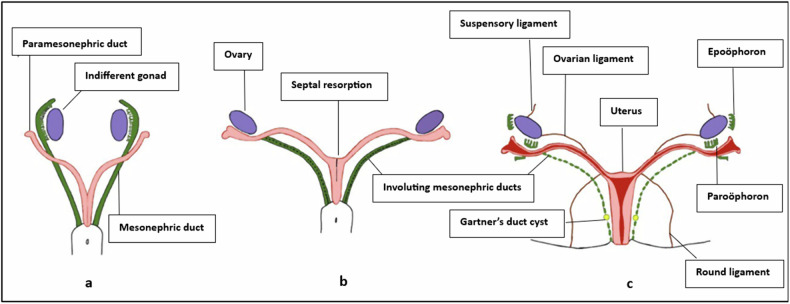
Development of female internal genitalia. **a** Indifferent gonads with undifferentiated genital ducts—Mullerian/paramesonephric ducts lying lateral to mesonephric ducts at the cranial end and medial to them at the caudal end. **b** In female fetuses, mesonephric duct involution occurs gradually with Mullerian duct fusion and septal resorption eventually. **c** Uterus, cervix, and vagina with supports of the uterus and mesonephric remnants (epoöphoron, paroöphoron, and Gartner’s duct cyst)

Development of female internal genitalia from Mullerian ducts occurs between 6 weeks and 11 weeks in utero. This process occurs in three stages [5] (Fig. 1):

### A) Duct development

Both male and female embryos begin with undifferentiated gonads and paired genital ducts, mesonephric (Wolffian) and paramesonephric (Mullerian). Differentiation of gonads depends on the Y chromosome. In the presence of the sex-determining region Y gene (sex-determining region Y gene) gene on Y-chromosome, testes develop in male fetuses. Sertoli cells in the testis produce Mullerian inhibiting hormone (MIH), which causes degeneration of Mullerian ducts. Testosterone, the male sex hormone, is produced by Leydig cells and is responsible for the development of Wolffian ducts into male reproductive organs (prostate, seminal vesicles). In the absence of a Y chromosome in female fetuses, the gonads differentiate into ovaries. After 6 weeks of gestation, due to the absence of MIH and under the influence of maternal and placental estrogens, Mullerian ducts develop and Wolffian ducts degenerate. Interruption at this stage results in developmental anomalies such as agenesis or hypoplasia of the uterus, fallopian tubes, cervix, upper vagina, and unicornuate uterus.

### B) Duct fusion

Eventually, the caudal Mullerian ducts fuse from the caudal to the cranial end, forming the uterus, cervix, and upper two-thirds of the vagina (Mullerian vagina) with the presence of an intervening septum initially. The lower one-third of the vagina is derived from the urogenital septum, although some debate exists on the dual origin of the vagina [6, 7]. The unfused cranial portion of ducts form fallopian tubes. Interruption at this stage results in fusion anomalies such as uterus didelphys (complete failure) or bicornuate (incomplete failure).

### C) Septal reabsorption

Finally, between 9 weeks and 12 weeks of gestation, degeneration of the fused margins of Mullerian ducts occurs along with vaginal plate canalization. Interruption at this stage results in resorption anomalies of the uterus (septate and arcuate uterus). In the vagina, failure of resorption of fused margins of Mullerian ducts results in the longitudinal vaginal septum (LVS), and failure of vaginal plate canalization results in the transverse vaginal septum. The process of development is generally completed by 20 weeks of gestation.

Since embryologically, ovaries develop from the primordial germ cells which migrate to the genital ridge, ovarian anomalies are not associated with MDAs.

## Imaging modalities

### Hysterosalpingography (HSG)

HSG is the initial tool for the evaluation of tubal patency in infertility. The role of HSG in the evaluation of MDAs is however limited because it does not provide any information about the external contour of the uterus or the uterine cavity not communicating with the cervix. It cannot be performed in agenesis or dysgenesis of the cervix [8].

### Ultrasound (USG)

USG is the most widely used imaging modality in obstetric and gynecological evaluation. 2D USG has limitations in terms of inadequate visualization of uterine contour, inter-operator variability, and low sensitivity. Currently, 3D transvaginal USG (3D TVS) is used for better assessment of external fundal contour and internal indentation of the endometrial cavity. Thick and echogenic endometrium in the secretory phase helps in better evaluation of MDAs, thus pelvic USG for their evaluation should be scheduled in the latter part of the menstrual cycle. Ovaries should be identified in all patients and malpositioned ovaries should be looked for, which would require a transabdominal scan at times.

### Magnetic resonance imaging (MRI)

MRI is the ideal imaging modality for accurate diagnosis of MDAs as it gives exquisite information regarding both external contour and internal anatomy. T2WI helps in the accurate delineation of uterine zonal anatomy and the presence of functional endometrium and helps in the precise classification of anomalies [9]. It also provides information regarding ovaries and adnexa. T1WI allows the identification of glands in endometriosis or blood products in hematometrocolpos [10]. Associated ovarian maldescent (high-placed ovaries at or above the level of internal iliac artery bifurcation) and renal anomalies can also be simultaneously identified.

In addition, the use of 3D TVS is not possible in patients who are not sexually active or those with vaginal agenesis, obstruction, and complex anomalies, MRI is best suited for accurate diagnosis of MDAs in these patients also.

#### MR imaging protocol

##### Patient preparation

MRI for evaluation of MDAs can be scheduled irrespective of the patient’s menstrual cycle. Some centers recommend fasting for 3–6 h, however, no consensus exists. Emptying the bladder 1 h prior to the examination is recommended to achieve a moderately filled bladder at the time of the scan. Unless contraindicated, antiperistaltic agents (20 mg of butylscopolamine or 1 mg glucagon IM/IV) are administered to reduce bowel motion artifacts and thus obtain high-quality MR imaging. Aqueous vaginal gel (~60 mL) can be administered in a small percentage of patients when required.

##### MRI acquisition

MRI pelvis is performed in the head-first supine position with free breathing using a dedicated phased-array pelvic coil. First, a three-plane localizer is acquired for planning the sequences. Among the sequences, the T2 sagittal sequence is acquired first. Oblique coronal sequences are planned parallel to the endometrial cavity and oblique axial sequences are planned perpendicular to the endometrial cavity. Whenever cervical anomalies are suspected, cervix-based coronal and axial sequences can be planned parallel and perpendicular to the cervical canal, respectively. T2 coronal SSFE sequences of the abdomen are done for evaluation of associated genitourinary anomalies (breath-holding/navigator-triggering). The order, acquisition parameters, and rationale of routine MRI sequences for the diagnosis of MDA along with a few optional sequences are described in Table 1 [1].

**Table 1:** Routine and additional MRI sequences for MDA diagnosis

Routine MRI sequences for MDA diagnosis									
Sequence	Imaging plane	FOV		TR, (ms)	TE, (ms)	Flip angle, (degrees)	NSA	Slice thickness/gap	Utility
2D T2W TSE/FSE*	a) True sagittal b) Oblique coronal (parallel to endometrium) c) Oblique axial (perpendicular to endometrium)	Entire pelvis Small (covering pelvic organs) Small (covering pelvic organs)		3000–4000	90–120	130–150	2	3–4/0	Evaluation of pelvis and planning of oblique axial and coronal sequences. Evaluation of external fundal contour and cavity shape in coronal and axial planes. The coronal plane is most critical in uterine malformation classification.
T1W TSE without and with fat suppression (FS) Or	True axial	Entire pelvis		400–600	15–25	130	2–3	3/0	Evaluation of blood products in obstructive anomalies and endometriosis.
T1W volume interpolated GRE Dixon	True axial	Entire pelvis		2.5–3.5	1–5	10–15	1	3/0	Shorter sequence, and thinner slices, and is excellent for the evaluation of blood products.
T2W SSFSE	True coronal	Large including abdomen (to cover kidneys)		1000–1600	80–90	130–150	1–2	5/0	Presence, position, and morphology of kidneys. Ectopic ovaries detection.
**Optional/additional MRI sequences for MDA diagnosis**									
**Sequence**			**Advantages**						
3D FS T2W			Provide submillimetre section thickness with multiplanar reformatting.						
Cervix-oriented sequences (oblique axial and coronal)			Accurate diagnosis of cervical anomalies.						
DCE FS T1W volume interpolated GRE			Characterization of additional/incidentally identified disease.						
CE FS T1W volume-interpolated GRE (coronal including kidneys)			Delayed imaging for assessment of suspected anomalous distal ureteric insertion.						

*DCE* dynamic contrast enhancement, *FSE* fast spin echo, *FOV* field of view, *SSFSE* single-shot FSE, *TSE* turbo spin echo

## Classification

The ESHRE/ESGE 2013 edition and the ASRM 2021 edition are the most commonly used classification systems.

ASRM 2016 (updated from the previous 1988 American Fertility Society classification system) classified Mullerian anomalies based on “failure of development” into seven categories/classes with similar clinical manifestations. ASRM was subsequently modified in 2021 to standardize terminology, ease identification in scientific databases, educate and facilitate use by providers, and promote patient awareness. The 2021 modification took away the classes, describes anomalies involving the uterus, cervix, and vagina, and divides them into nine descriptive categories [3]. The nine categories include:

1. Mullerian agenesis
2. Cervical agenesis
3. Unicornuate uterus
4. Uterus didelphys
5. Bicornuate uterus
6. Septate uterus
7. Longitudinal vaginal septum (LVS)
8. Transverse vaginal septum
9. Complex anomalies

Mullerian anomalies represent a continuum of development and thus, some anomalies (particularly vaginal) may be seen in more than one category. In addition, ASRM 2021 also includes specific diagnostic elements for septate, arcuate, and normal uteruses with differentiation of normal and arcuate uterus. Categories of Mullerian anomalies can be assessed by any imaging technique such as 2D/3D USG, MRI, sonohysterogram, hysteroscopy (combined with imaging of external uterine contour), or combined hysteroscopy–laparoscopy.

**Fig. 2:**
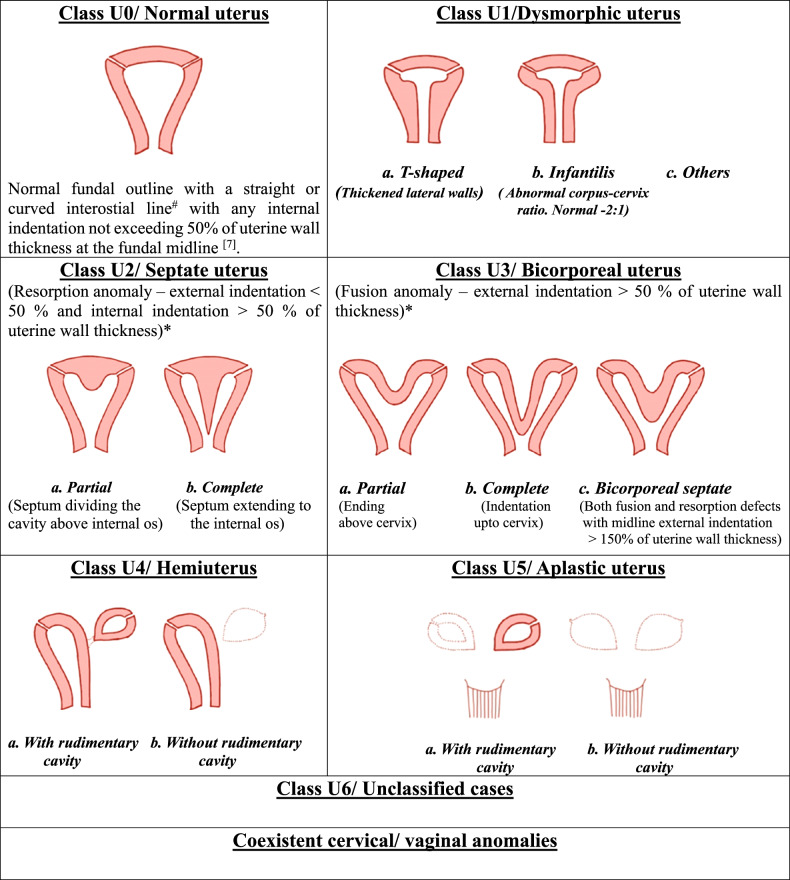
ESHRE/ESGE classification of Mullerian anomalies. ^#^The interostial line connects the uterine ostia of the fallopian tubes. ^*^Uterine fundal cleft or external indentation is used to differentiate fusion anomalies (didelphys and bicornuate) from resorption anomalies (septate and arcuate), which can be precisely demonstrated on a true coronal image [10]. Fundal cleft > 1 cm or external indentation > 50% of uterine wall thickness suggests fusion anomalies, whereas < 1 cm or external indentation < 50% of uterine wall thickness represents resorption anomalies [10]

ESHRE/ESGE classification system is primarily based on anatomical deviation, due to similar embryological origin [11]. 3D USG is the main diagnostic modality used to classify anomalies and instead of using absolute measures, relative thickness/ratios are used (Fig. 2 and Table 2).

**Table 2:** Coexistent cervical/vaginal anomalies

C0—Normal cervix	V0—Normal vagina
C1—Septate cervix	V1—Longitudinal non-obstructing vaginal septum
C2—Double cervix	V2—Longitudinal obstructing vaginal septum
C3—Unilateral cervical aplasia	V3—Transverse vaginal septum and/or Imperforate hymen
C4—Cervical aplasia	V4—Vaginal aplasia

In this article, MDAs are discussed based on ASRM classification, and differences with ESHRE/ESGE classification are highlighted when relevant.

### Normal uterus

**Fig. 3:**
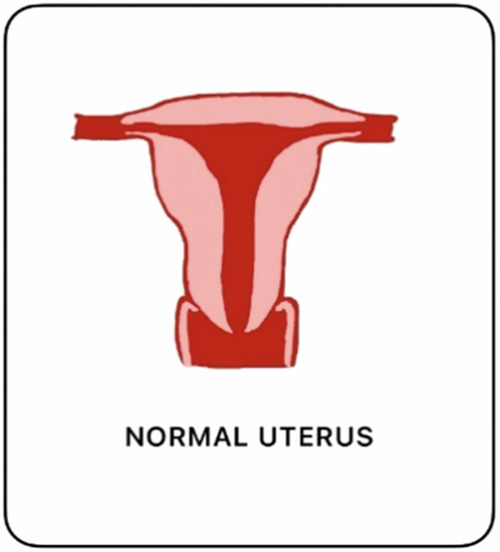
Diagrammatic representation of normal uterus

Normally, the uterus is pear-shaped [12]. According to ASRM 2021, the normal uterus has a convex or straight external fundal contour with no internal indentation (Fig. 3).

### Mullerian agenesis

#### General information

**Fig. 4:**
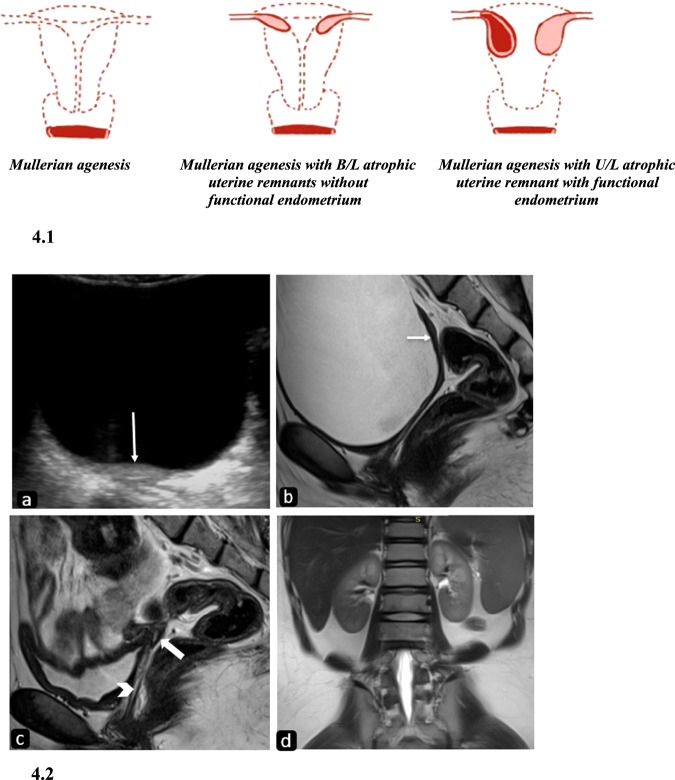
Diagrammatic representation and imaging features of Mullerian agenesis. 4.1: Diagrammatic representation of variants of Mullerian agenesis. 4.2: MRKH syndrome type 1: (**a**) USG pelvis of a seventeen-year-old girl 46XX karyotype in the transverse plane reveals an ovoid hypoechoic structure posterior to the urinary bladder with poor zonal anatomy (arrow) suggesting the possibility of a hypoplastic uterus. Sagittal T2W MR image (**b**) reveals a small hypointense linear structure posterior to the bladder in the expected location of the uterus (arrow). The sagittal image obtained after partially emptying the bladder (**c**) better depicts the rudimentary zonal anatomy confirming the presence of a hypoplastic uterus (thick arrow). Also, note the vagina seen as a linear hyperintensity between the bladder and rectum (thin arrow). T2W coronal image (**d**) of the upper abdomen reveals both kidneys to be normal

Failure of the early development of Mullerian ducts results in the agenesis of fallopian tubes, uterus, cervix, and proximal two-thirds of the vagina in variable degrees (Fig. 4). This occurs as a part of Mayer–Rokitansky–Kuster–Hauser (MRKH) syndrome with a prevalence of 1 in 4500 women. It is characterized by Mullerian agenesis, normal development of secondary sexual characteristics, and a normal 46XX karyotype. MRKH is of two types: type I or isolated uterovaginal aplasia and type II MRKH, which is characterized by incomplete aplasia and/or associated renal (40%), cardiac, skeletal (30–40%) abnormalities and hearing defects (10–25%) [13]. Thus, it is generally referred to as Mullerian duct aplasia-renal agenesis-cervicothoracic somite dysplasia association and is more common (Fig. 4).

#### Imaging

HSG cannot be performed in Mullerian agenesis and thus has no role in diagnosis. USG is the initial imaging modality, and findings include the absence of a uterus and identification of uterine remnants, if present, may be challenging. Normal-appearing ovaries are seen in either a normal or abnormal location.

Sagittal T2W MR images are especially useful as the expected location of the uterus may be indicated from the location of the bladder, urethra, and lower vagina [1]. There may be a complete absence of uterine tissue, or more often, a small hypoplastic uterus may be seen. Pre-pubertal females show no zonal anatomy, whereas, after puberty, poorly defined zonal anatomy may be seen under the influence of female hormones. When bilateral, atrophic uterine remnants are seen as solid ovoid or elongated structures exhibiting hypo to isointense signal on T1WI. On T2WI, the presence of functional endometrium is suggested by cavitation of uterine buds which appears as a target pattern consisting of central hyperintense endometrium surrounded by intermediate signal of junctional zone and medium to high signal intensity of myometrium [6]. The uterine remnants may show connecting or converging fibrous bands.

Normally, the vagina appears as a T2 hypointense structure between the urethra, neck of urinary bladder anteriorly, and rectum posteriorly, which can be well appreciated on sagittal and axial T2W images. Outer T2 hypointense muscular and fibrous tunica contrast with inner T2 hyperintense mucosa and mucus-filled vaginal lumen. Sagittal T2WI is useful to measure the maximum vaginal length and thus aid in vaginal dilatation and surgical procedures [6].

#### Clinical importance and management

These patients clinically present with primary amenorrhea and pelvic pain in the presence of functional endometrium in the uterine remnants. Pain can be cyclical due to functional endometrium or chronic due to retrograde menstruation causing endometriosis.

**Fig. 5:**
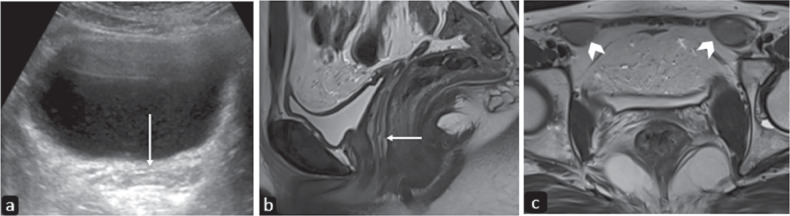
Imaging features of androgen insensitivity syndrome. USG image in the transverse plane (**a**) of a seventeen-year-old phenotypic female with primary amenorrhea and 45XY karyotype reveals a linear echogenic structure posterior to the bladder likely vaginal canal with no evidence of any uterine tissue or ovaries. T2 weighted sagittal MR image (**b**) demonstrates a linear hyperintense vaginal canal (arrow) between the urinary bladder anteriorly, rectum, and anus posteriorly. Note the absence of a cervix or uterus. T2W axial image at the level of the inguinal canal (**c**) shows bilateral testes with epididymis seen in the inguinal region (arrowheads)

Imaging, especially MRI, can help in the differentiation of Mullerian agenesis from other entities presenting with primary amenorrhea and with normal secondary sexual characteristics [13] (Table 3 and Fig. 5).

**Table 3:** Differential diagnosis of primary amenorrhea

Entity	Differentiating feature from Mullerian agenesis/MRKH
Androgen insensitivity syndrome	Abdominal or inguinal testes.
WNT defects	Hyperandrogenism in normal phenotypic females.
Mullerian derivative aplasia	Abnormal karyotype (no imaging differences).
Mc Kusick–Kaufmann syndrome	Hydrometrocolpos due to isolated vaginal atresia, postaxial polydactyly, and congenital heart malformations.
Other obstructive Mullerian anomalies (transverse vaginal septum, imperforate hymen, and cervical agenesis)	Imaging appearances as described further in this paper.

Treatment of Mullerian agenesis includes vaginal dilatation and the creation of a neovagina by surgical procedures to enable normal sexual function.

### Cervical agenesis

#### General information

**Fig. 6:**
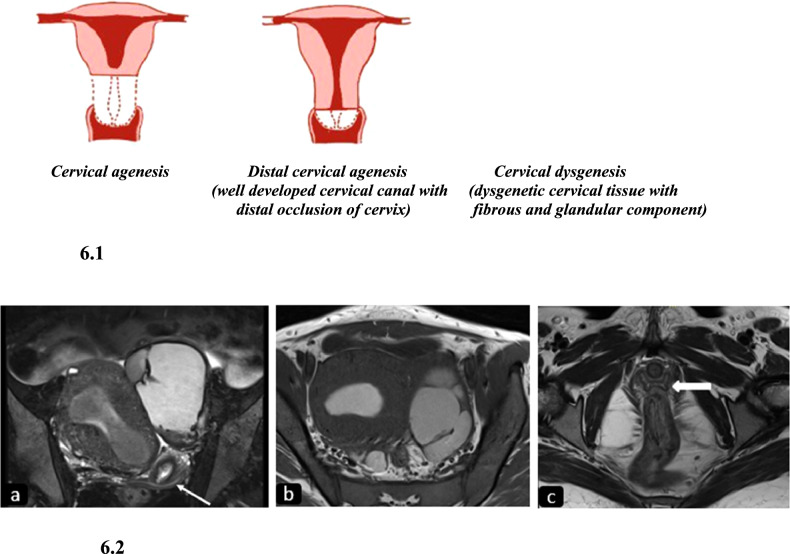
Diagrammatic representation and imaging features of cervical agenesis. 6.1: Diagrammatic representation of variants of isolated cervical agenesis. Variants can have variable cervical length. 6.2 Isthmic agenesis: T2W coronal (**a**) and T1W axial (**b**) images of a nineteen-year-old girl with primary amenorrhea (**a**) reveal a normal-sized uterus with the endometrial cavity distended by T2 hypointense and T1 hyperintense contents suggesting blood products. Note the cervix (thin arrow) in **a** with no communication between the uterus and cervical canal. The uterus is pushed to the right by a hemorrhagic left tubo-ovarian mass due to retrograde menstruation. The normal H-shaped vagina (thick arrow) is well seen on a more inferior T2W axial image (**c**)

Cervical agenesis is a part of Mullerian agenesis. Isolated cervical agenesis is extremely rare and results from interrupted development of Mullerian ducts with no communication between the uterus and vagina (Fig. 6).

#### Imaging

USG findings include: a lack of a well-defined cervical canal with an obstructed and distended endometrial cavity, fallopian tubes with blood contents, and endometriosis. MRI is the most useful imaging modality as the normal cervix is clearly identified. On T1WI, the normal cervix exhibits intermediate signal intensity with poor delineation of zonal anatomy. On T2WI, the cervix shows four zones from outside which include: a central hyperintense mucus-filled cervical canal surrounded by high-signal endocervical mucosa and glands, hypointense fibrous stroma, and outer intermediate signal intensity of loose stroma [14]. In cervical agenesis, no cervical tissue or dysgenetic cervix is visualized between the uterus and vagina. Additional findings of retrograde menstruation such as hematometra, hematosalpinx, or endometrioma are seen in the form of distended fluid-filled uterine cavities and fallopian tubes which appear hyperintense on T1WI and heterogeneous on T2WI [15].

#### Clinical importance and management

The clinical presentation is similar to other obstructive Mullerian anomalies i.e., primary amenorrhea and pelvic pain due to hematometra, hematosalpinx, and endometriosis. Treatment options include medical and surgical procedures such as cervicoplasty and uterovaginal anastomosis. In case of failure of these procedures, a hysterectomy of the obstructed uterus may be required [16].

### Unicornuate uterus

#### General information

Unicornuate uterus is formed due to the normal development of one Mullerian duct and failed or arrested development or failure of migration of the other. The prevalence of this anomaly is 0.3%, comprising 3–13% of all Mullerian defects [17]. The uterine remnants can be atrophic without endometrium or functional with endometrium within (cavitatory) (Fig. 7.1). It is the most common MDA associated with renal anomalies (40%); renal agenesis is the most frequent and is ipsilateral to the rudimentary horn [18].

**Fig. 7:**
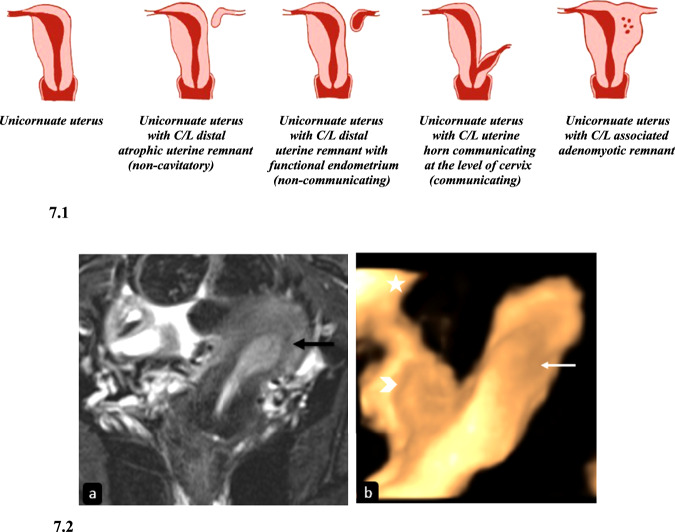
Diagrammatic representation and imaging features of unicornuate uterus. 7.1: Diagrammatic representation of variants of unicornuate uterus. 7.2. Unicornuate uterus. T2-FS coronal MR image (**a**) in a 31-year-old female with primary infertility reveals an off-midline left-sided banana-shaped uterine horn (black arrow) continuous with the cervix inferiorly s/o left-sided unicornuate uterus. 3D transvaginal USG image (**b**) in another patient shows a left-sided unicornuate uterus (white arrow) with a small non-cavitary non-communicating rudimentary horn on the right side (arrowhead). The right ovary is normal (asterisk)

#### Imaging

HSG has a limited role in the diagnosis of the contralateral uterine remnant as it can only detect the cavitatory, communicating uterine remnant. Also, a bicornuate bicollis uterus and uterus didelphys can give a similar appearance when only one of the cervical canals is cannulated. On USG, it is seen as a small, oblong-shaped, off-midline structure with endometrium, however, a rudimentary horn may be missed or misdiagnosed as pelvic mass or cervix [1]. On MRI, the unicornuate uterus is seen as a small, curved banana-shaped uterus with normal width of endometrium and myometrium [19]. The contralateral uterine remnant and its communication with the unicornuate uterus is also well-visualized. Detection of endometrium within the uterine remnant is important as the clinical presentation depends on the same. Diffuse low signal intensity on T2WI suggests the absence of endometrium, whereas the presence of zonal anatomy suggests cavitatory type. The cavitatory, non-communicating type manifests with blood products distending the endometrial cavity and findings of endometriosis (Fig. 7.2).

A unicornuate uterus with a cavitatory communicating remnant can be distinguished from a bicornuate uterus by the asymmetry of two uterine cavities in the former, which is extremely rare in the latter.

#### Clinical importance and management

The age and presenting symptoms depend on the status of the rudimentary horn. The cavitatory, communicating rudimentary horn type presents with malpresentation, preterm labor, or pregnancy loss. The cavitatory, non-communicating rudimentary horn type presents with lateralized cyclical pelvic pain [20]. Simple and atrophic uterine remnant types are usually detected incidentally. Treatment options include resection or reunification of the uterine remnant with unicornuate uterus.

### Uterus didelphys

#### General information

Uterine didelphys is a fusion anomaly that arises due to complete or incomplete arrest of fusion of Mullerian ducts, resulting in duplication of uterine horns and cervix (Fig. 8.1). Duplication of the proximal vagina can also be seen. It is reported in 0.1–0.5% of women, comprising ~11% of uterine malformations [21]. Duplicated or double vagina is the result of LVS, either complete (75%) or partial. Renal agenesis is most commonly associated with this MDA occurring in almost 20% of cases, uterine didelphys with obstructed hemivagina with ipsilateral renal agenesis (OHVIRA) being the most common association [22].

**Fig. 8:**
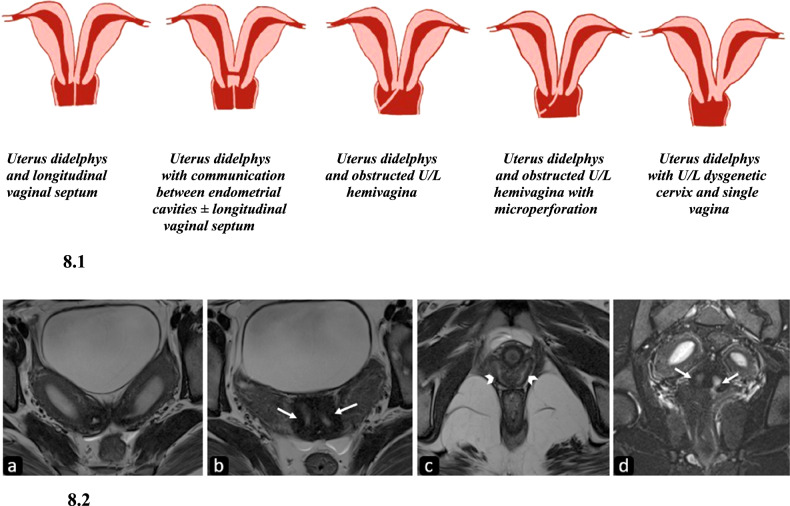
Diagrammatic representation and imaging features of uterus didelphys. 8.1: Diagrammatic representation of variants of uterus didelphys. 8.2. Uterus didelphys with double cervix and vaginal septum. Axial T2-weighted (**a**–**c**), and T2-FS coronal (**d**) MR images in a twenty-year-old female patient demonstrate two widely divergent uterine cavities showing normal zonal anatomy with no communication between them (**a**). Two cervical canals (arrows in **b**, **d**) are seen separated by an intervening myometrium suggesting a double cervix giving an owl’s eye appearance with increased intercervical distance. Two vaginal canals (arrowheads) are better appreciated in (**c**)

#### Imaging

HSG shows two separate cavities, cervical canals, and often a double vagina [8]. In case of obstructed hemivagina, it may be misdiagnosed as a unicornuate uterus. In didelphys, two symmetrical widely divergent, non-communicating endometrial cavities are seen and two cervical canals and a double upper vagina should be looked for.

MRI demonstrates two separate divergent uterine cavities of normal volume with normal zonal anatomy. The double cervix is seen as two separate cervical canals with a mean intercervical distance of approximately 12.05 mm, significantly higher than that of the septate cervix in bicornuate and septate uteri [23]. In obstructed hemivagina with hematometrocolpos, T1 hyperintense blood products are seen within [19] (Fig. 8.2).

#### Clinical importance and management

Patients with hemivaginal obstruction present with dysmenorrhea due to endometriosis, pelvic infections, and adhesions, and those without obstruction are usually asymptomatic. Treatment includes resection of obstructing vaginal septum or removal of obstructed hemiuterus.

### Bicornuate uterus

#### General information

**Fig. 9:**
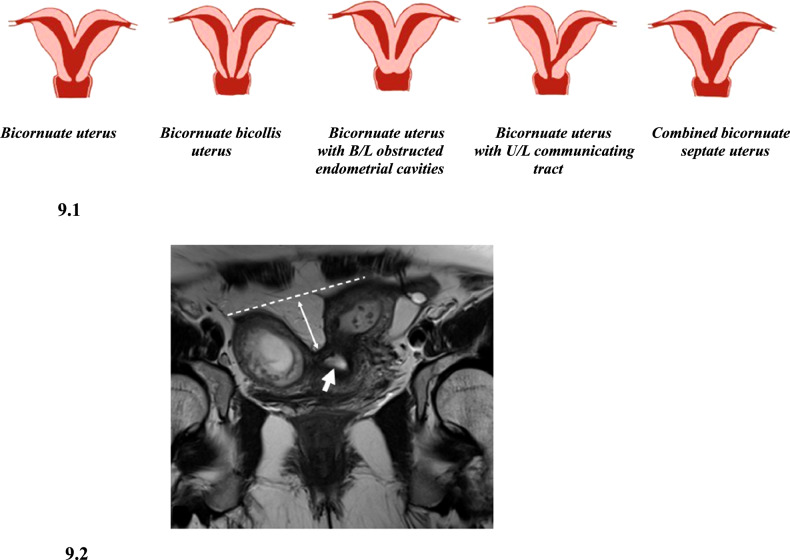
Diagrammatic representation and imaging features of bicornuate uterus. 9.1: Diagrammatic representation of variants of bicornuate uterus. 9.2. Bicornuate uterus with single cervix. T2 weighted coronal MR image demonstrates two widely separate endometrial cavities with external indentation greater than 1.5 cm (double-edged arrow) with a single cervix (thick arrow)

Impairment in the fusion of Mullerian ducts results in a bicornuate uterus resulting in a heart-shaped uterus [12]. It is seen in 0.1–0.6% of women, comprising 10% of Mullerian anomalies [1]. The cervix can be single (unicollis), septate/double (bicollis), or may show unilateral cervical atresia (Fig. 9). Vagina may be single or have a longitudinal or transverse vaginal septum either obstructing or with microperforation.

#### Imaging

On HSG, differentiation of the bicornuate uterus from the septate uterus is difficult as external fundal contour cannot be assessed, but the bicornuate uterus demonstrates two separate uterine cavities with an intercornual angle of > 105 degrees in contrast to the septate uterus with an intercornual angle < 75 degrees [8]. 2D USG is not reliable as the external fundal contour cannot be assessed. On 3D USG, deep fundal cleft >1 cm (ASRM) and diverging endometrial cavities are seen. MRI findings include a deep fundal cleft, two uterine cavities of normal volume with normal zonal anatomy, and an intercornual distance >4 cm. The intervening tissue at the level of the fundus shows T2 hyperintense myometrium and at the level of the lower uterine segment may show myometrium or T2 hypointense fibrous tissue [19]. In bicornuate unicollis, a single cervical canal is seen, while in bicornuate bicollis, two T2 hyperintense cervical canals (duplicated cervix) are seen separated by intervening myometrium giving the “owl eye” appearance [24]. The intracervical distance in the bicornuate uterus according to a study by Smith et al is ~5.4 mm, less than that in uterus didelphys [23]. The vagina may show a LVS (in one-fourth of cases) which, if present, makes bicornuate bicollis virtually indistinguishable from uterus didelphys.

This type of anomaly is referred to as the bicorporeal uterus in the ESHRE/ESGE classification system (see Fig. 3 for details).

#### Clinical importance and management

Patients may present with menstrual symptoms such as dyspareunia, lateralized cyclical pain, and menstrual leakage even with tampon use or pregnancy concerns such as preterm labor, malpresentation, pregnancy loss, and post-partum hemorrhage. Surgical management includes reunification by metroplasty (Strassman/Jones/Tompkins).

### Septate uterus

#### General information

**Fig. 10:**
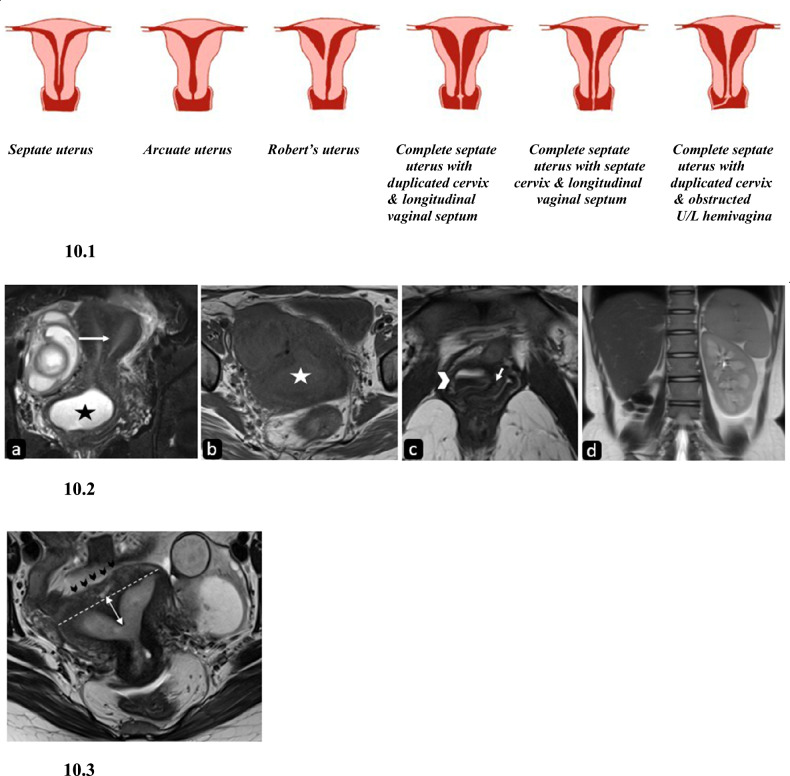
Diagrammatic representation and imaging features of septate uterus. 10.1: Diagrammatic representation of variants of septate uterus. 10.2. Complete septate uterus with obstructed hemivagina and ipsilateral renal agenesis. T2 weighted coronal and T1 weighted axial MR images (**a**, **b**) in a twenty-one-year-old female patient demonstrate two endometrial cavities separated by a septum (arrow) extending into the cervix and vagina, the right hemivagina is distended with T2 hyperintense (black asterisk in **a**) and mild T1 hyperintense contents (white asterisk in **b**) suggesting blood products. To advantage, T2 weighted axial MR image (**c**) depicts the two vaginal canals separated by a longitudinal septum (short arrow) with layering of blood products as dependent hypointense and upper hyperintense contents (arrowhead) in distended right hemivagina. T2 weighted coronal MR image of the upper abdomen (**d**) reveals the absence of the right kidney. 10.3. Partial septate uterus. Axial T2 weighted MR image of a twenty-six-old year female patient with recurrent first-trimester pregnancy loss demonstrates a straight external fundal contour (black arrowheads) with internal indentation greater than 1.5 cm (double-headed arrow) from the interostial line (dashed line). The upper part of the septum is isointense to the adjacent myometrium suggesting the muscular septum does not reach up to the internal os. Note made of a tubo-ovarian hemorrhagic mass in the left adnexa

Failure of resorption of uterovaginal septum results in resorption anomalies, i.e., septate and arcuate uterus. Septate uterus is the most common Mullerian anomaly, contributing to ~55% of MDA, and has a prevalence of 2.3% [25]. It is associated with high rates of both first and second-trimester abortions, preterm deliveries, and fetal malpresentation. However, there is no definite consensus on the direct relationship between the septate uterus and infertility [10]. Septae can be partial or complete depending on whether it is reaching the internal os or not (Fig. 10). The cervix may be single, septate, or duplicate (the latter being very rare), and the vagina may show a non-obstructing septum or obstructed hemivagina (OHVIRA). The septate uterus may also be uncommonly associated with OHVIRA syndrome.

#### Imaging

On HSG, an intercornual angle < 75° suggests a septate uterus rather than a bicornuate uterus, however, the external uterine contour cannot be demonstrated and thus is not reliable.

On MRI, a convex or straight external fundal contour with an intervening septum is seen. The septum may be fibrous (low T2 signal intensity) or muscular (intermediate signal intensity) and is seen separating the endometrial cavities which are reduced in volume and show normal intercornual distance (2–4 cm), normal endometrial to myometrial width and ratio, but internal indentation > 1.5 cm (ASRM) suggests septate uterus. On the other hand, internal indentation ≥ 1 cm to ≤ 1.5 cm is considered a “gray zone” according to ASRM criteria [10]. The septum can be partial (not reaching up to the internal os) or complete (reaching up to the internal os) and may also extend into the cervix or vagina. In a complete septate uterus with a septate cervix, the intercervical distance is approximately 5.43 mm [23]. Rarely, true cervical duplication can also be seen.

At times, external indentation of less than or equal to 1 cm is seen in the uterus with internal indentation satisfying the criteria of septate uterus. This is known as a hybrid septate uterus, an entity under combined fusion and resorption anomaly [26].

Another clinically insignificant milder fusion anomaly that should be distinguished from the septate uterus is the arcuate uterus. It has no significant effect on pregnancy outcomes. Internal indentation < 1 cm (ASRM) with the angle of leading edge > 90° suggests an arcuate uterus. The indentation in the arcuate uterus is broad and saddle-shaped (Fig. 11.2). No fibrous tissue or septum is demonstrated in the arcuate uterus [1, 18].

**Fig. 11:**
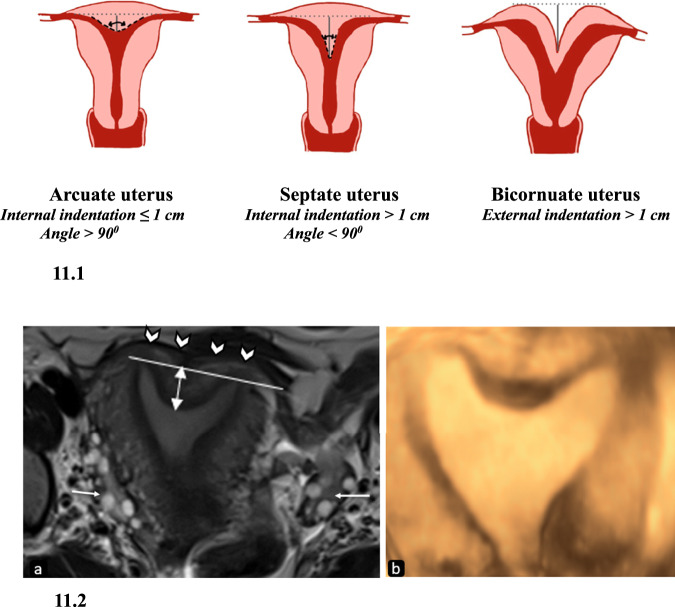
Differences between arcuate, septate, and bicornuate uteri with imaging features of the arcuate uterus. 11.1: Diagrammatic representation of differences between arcuate, septate, and bicornuate uteri. 11.2. Arcuate uterus: axial T2W MR image (**a**) of an arcuate uterus in a 26-year-old female patient with recurrent pregnancy loss shows a normal external fundal outline (white arrowheads) with an internal indentation lesser than 1.5 cm (double-headed arrow) from the interostial line. Note normal ovaries bilaterally (arrows). **b** 3D transvaginal USG image shows a broad saddle-shaped indentation of the arcuate uterus

The criteria for septate uterus according to ESHRE/ESGE classification are depicted in Fig. 2. Most significantly, there is no category of arcuate uterus in this classification and a number of them are classified as septate uterus as per ESHRE/ESGE resulting in the overdiagnosis of septate uterus according to the experts, almost three-times increase as per Ludwin and Ludwin [4] (Fig. 11.1 and Table 4).

**Table 4:** Differentiating features between bicornuate and septate uterus

ASRM category	Bicornuate uterus	Septate uterus
External indentation	> 1 cm	No indentation or indentation < 1 cm
Internal indentation	–	> 1.5 cm
Morphology	Two well-formed uterine cornua with convex fundal contour in each [31]	Presence of septum that may(complete) or may not (partial) extend to the cervix [31]
Intercornual angle (HSG)	> 105°	< 75°
Intercornual distance (MRI)	Increased (> 4 cm)	Normal (2–4 cm)
Intervening tissue	Central myometrium of intermediate signal intensity	The septum can be fibrous (T2 hypointense) or muscular (intermediate T2 signal intensity)
Cervix	Duplicate or septate or single	Septate or single
Intercervical distance	5.4 mm	5.4 mm
Treatment	Metroplasty	Septal resection

#### Clinical importance and management

The clinical presentation depends on the type of septum; muscular septum is more vascular and predominantly presents with changes in uterine motility resulting in preterm delivery or miscarriage. The fibrous septum is less vascular and interferes with implantation [10]. It should be distinguished from Ashermann syndrome where multiple T2 hypointense intrauterine synechiae are seen. Treatment includes hysteroscopic incision or resection of fibrous septum and transabdominal approach for muscular septum. The absence of a separate class for arcuate uterus in the ESHRE/ESGE classification system and the use of uterine wall thickness results in its misdiagnosis as a septate uterus which renders the patient eligible for septal resection. Thus, it has been suggested that ESHRE/ESGE criteria must not be used for the diagnosis of septate uterus, and usage of uterine wall thickness as a reference value in the detection of internal and external structural distortions must be discontinued [4].

Another important consideration is that the septate uterus must be distinguished from the bicornuate uterus, as the treatment of these differs greatly, the latter is treated by laparoscopic metroplasty while the former is by septoplasty as discussed earlier. MRI is an excellent tool to differentiate these two entities by giving information about external fundal contour as well which is extremely important to distinguish between the two (Fig. 11.1 and Table 4).

## Developmental vaginal anomalies

### Transverse vaginal septum

#### General information

Incomplete vertical fusion between the vaginal components of the Mullerian tubercle and urogenital sinus results in the formation of transverse vaginal septum. It is one of the rare anomalies of the female reproductive tract with an incidence of 1 in 30,000–84,000 women [27]. The septum can be low (15%)/mid (40%)/high (45%) ± microperforation (Fig. 12.1). The thick septum can be so thick as to extend from the upper to lower portion of the vagina resulting in the formation of a small upper vaginal cavity and small lower residual vagina appearing as a dimple/flush with the introitus known as distal vaginal atresia/agenesis. It should be differentiated from imperforate hymen and cervical dysgenesis as they also present with primary amenorrhea and hydrometrocolpos. Imperforate hymen occurs due to failure of recanalization of the inferior most vaginal membrane. It is seen as a bulging, bluish membrane that transilluminates at the level of the introitus and rarely requires imaging.

**Fig. 12:**
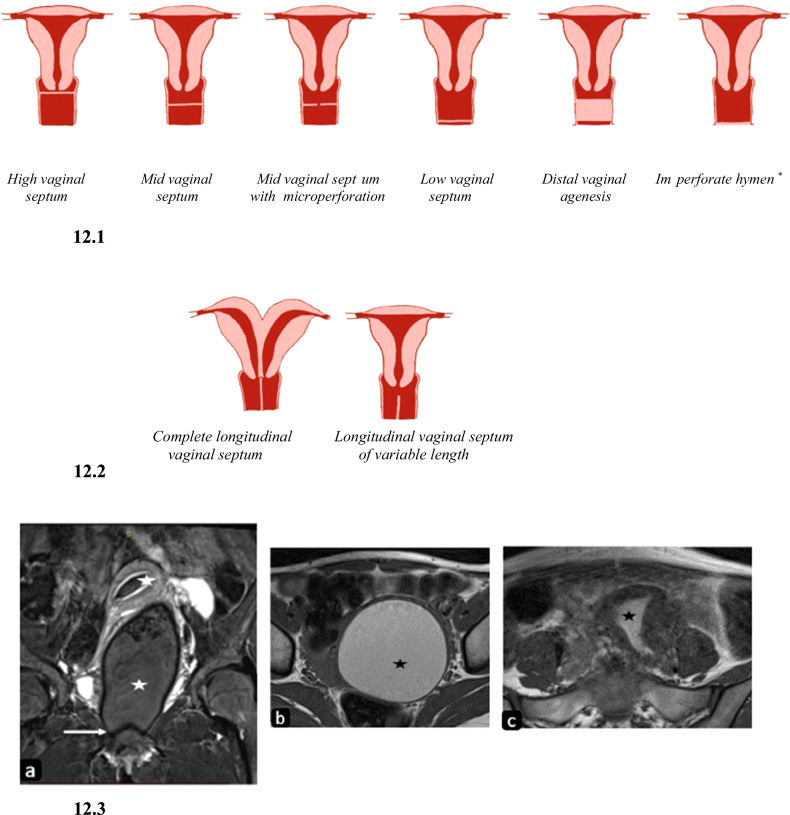
Diagrammatic representation and imaging features of developmental vaginal anomalies. 12.1: Diagrammatic representation of variants of the transverse vaginal septum and imperforate hymen*. 12.2: Diagrammatic representation of longitudinal vaginal septum. 12.3: Transverse vaginal septum. T2W coronal image (**a**) in a nineteen-year-old female with primary amenorrhea shows a hypointense linear structure at the inferior aspect of vaginal cavity (white arrow) with upstream distension of vagina and endometrial cavity showing heterogeneously hypointense contents (white asterisks) within which appear hyperintense (black asterisks) on T1WI axial images (**a**) and (**b**) suggestive of hematometrocolpos

Associations include uterine (didelphys, septate, and bicornuate) and genitourinary anomalies such as imperforate hymen, imperforate anus, ectopic ureter, and vesicovaginal fistula [28].

#### Imaging

USG and MRI demonstrate obstructing septum and resultant hydrometrocolpos. At MRI, there is an abrupt cutoff of the caliber of the vagina with upstream hydrocolpos or hematocolpos (Fig. 12.3). A normal vagina below the septum cannot always be identified, but if present, it helps in differentiating from complete vaginal atresia [29].

#### Clinical importance and management

Patients usually present with obstructive symptoms. Septal resection is the treatment of choice.

### Longitudinal vaginal septum (LVS)

#### General information

Failure of lateral fusion of the uterovaginal septum results in the formation of an LVS [10] (Fig. 12.2). It is seen in uterus didelphys, bicornuate bicollis, and complete septate uterus. Uterus didelphys is the most common anomaly associated with LVS, however, in clinical practice, LVS is most commonly seen with septate uterus due to its high prevalence. LVS can be complete or partial, central or eccentric, non-obstructing or obstructing ± microperforation [1].

#### Imaging

On USG, a hypoechoic band of fibrous tissue of variable length is seen extending through the vagina. MRI shows a T2 hypointense band along the length of the vagina resulting in a double vagina. The two vaginal cavities can be either symmetrical or asymmetrical. Aqueous vaginal gel can be applied for better visualization of the cavities and septum.

#### Clinical importance and management

The clinical presentation depends on the status of obstruction caused by the septum. Non-obstructing types are usually asymptomatic and may present with difficulty in tampon insertion, leakage even with tampon use, or laceration during intercourse. Patients with obstructing types present with symptoms of hemivaginal obstruction and those with microperforate vaginal obstruction present with abnormal uterine bleeding. These should be differentiated from imperforate hymen, vaginismus, and vaginal adhesions. Indications for resection of the septum include dystocia and dyspareunia [30].

### Complex anomalies

A combination of features of more than one class results in complex anomalies. Some possibilities include (a) agenesis of the uterine isthmus, (b) bicornuate uterus with U/L communicating tract and transverse vaginal septum, and (c) obstructed U/L hemivagina, hemiuterus, and single cervix with separate C/L patent hemiuterus, cervix, and vagina.

It is important that radiologists are aware of the similarities and differences between ASRM and ESHRE/ESGE classification and their impact on clinical management which have been summarized in Table 5 for convenience.

**Table 5:** Comparison of ASRM 2021 and ESHRE/ESGE 2013 classification systems

S.No	ASRM classification 2021		ESHRE/ESGE classification 2013	
	Nomenclature	Criteria	Nomenclature	Criteria
1.	Normal uterus	Convex fundus with no internal/external indentation	U0/ Normal uterus	Straight/ curved interostial line with internal indentation not exceeding > 50% uterine wall thickness at fundal midline.
2.	Arcuate uterus	Normal fundal contour with internal indentation^*^ < 1cm Angle of divergence^#^ > 90^o^	No category of arcuate uterus	-
3.	Septate uterus^α^	Normal or external indentation^§^ ≤ 1 cm Internal indentation > 1.5 cm Angle of divergence < 90^o^ Cervix – normal/ septate	U2/ Septate uterus	Normal outline/ external indentation < 50% uterine wall thickness with internal indentation > 50% uterine wall thickness at fundal midline.
4.	Bicornuate uterus^α^	External indentation > 1 cm Cervix – normal/septate/double cervix	U3a/ Partial bicorporeal uterus	External indentation > 50% uterine wall thickness at fundal midline partly dividing uterus corpus above the level of cervix (or internal os)
5.	Uterus didelphys^α^	Two separate uterine bodies with duplication of cervix	U3b/ Complete bicorporeal uterus	External indentation > 50% uterine wall thickness at fundal midline partly dividing uterus corpus upto the level of cervix (or internal os)
6.	Unicornuate uterus	Elongated uterine body (banana shaped) and deviated off-midline ± noncavitatory/cavitatory; non-communicating/ communicating rudimentary horn	U4/ Hemiuterus	Unilateral formed uterus, ± functional rudimentary cavity (4a/ 4b) [does not incorporate non-functioning rudimentary horn] [4]
7.	Mullerian agenesis	Absent or rudimentary uterus	U5/ Aplastic uterus	Absence of any fully or unilaterally developed uterine cavity
8.	Cervical agenesis		Coexistent class of cervical anomalies C0-C4	
9.	Transverse vaginal septum and longitudinal vaginal septum		Coexistent class of vaginal anomalies V0-V4	
10.	Complex anomalies		U6 – Unclassified anomalies	

^§^ -External indentation: Depth from interostial line to the indentation of uterine fundus at midline

^*^ -Internal indentation: Depth from interostial line to the lowest point of indentation of uterine cavities

^#^ -Angle of divergence or indentation: Angle formed by apex of endometrial cavity and the interostial line

^α^ -Vagina can be normal/septate—transverse or longitudinal

-cervical and vaginal anomalies are separately sub-classified in ESHRE/ESGE classification.

-Separate category of cervical agenesis, transverse and vaginal septae have been introduced in ASRM 2021 classification

-Sources- References [3, 4, 10]

## Conclusion

MDA is a broad spectrum of embryological anomalies of the female internal genitalia, which may manifest with diverse gynecological and obstetrical symptoms due to the anomalies themselves and their complications like endometriosis. MDAs such as Mullerian agenesis, and obstructive anomalies such as transverse vaginal septum cause primary amenorrhea, while the others such as unicornuate, bicornuate, and didelphys uteri affect reproductive outcomes causing malpresentation, preterm labor, and post-partum hemorrhage, etc. Septate uterus is the most common MDA. A fibrous septum interferes with implantation resulting in recurrent pregnancy losses, while a muscular septum restricts uterine motility resulting in preterm labor, however, the direct relationship between septate uterus and infertility has not been established [10]. A correct diagnosis and differentiation of arcuate from bicornuate uterus is important because septoplasty is known to improve clinical outcomes in the septate uterus. ASRM 2021 and ESHRE/ESGE classification systems are the two main classification systems available. However, in this context, it is important to remember that a septate uterus is overdiagnosed by the ESHRE/ESGE classification system due to the use of uterine wall thickness as the criterion and lack of an entity of arcuate uterus, thus leading to overtreatment of the septate uterus without expected benefits. Recommendations are to not use this classification for diagnosis of septate uterus till further modifications [4].

Currently, MRI is the imaging modality of choice in suspected MDAs as it not only accurately detects the anomaly and its associated complications but also aids in its classification helping in correct patient management. The emphasis, however, should be on the accurate description of a detected anomaly rather than attempting to rigidly fit the identified anomaly into one particular class, as the former is more clinically relevant. Assessed anomalies should be precisely communicated with the clinicians to aid in treatment. Once an MDA is identified, radiologists must remember to look for associated urinary tract, skeletal anomalies, and syndromic associations. Closely working with a multidisciplinary team of radiologists and gynecologists can greatly improve patient management.

## Data Availability

The data used during the current review are available from the corresponding author upon reasonable request.

## Abbreviations

ASRM: American Society of Reproductive Medicine
ESHRE/ESGE: European Society of Human Reproduction and Embryology and the European Society for Gynecologic Endoscopy
FSE: Fast spin echo
FS: Fat suppression
LVS: Longitudinal vaginal septum
MRKH: Mayer–Rokitansky–Kuster–Hauser
MDA: Mullerian duct anomalies
MIH: Mullerian inhibiting hormone
OHVIRA: Obstructed hemivagina with ipsilateral renal agenesis
